# Clinical risk factors associated with postoperative malnutrition in patients with laryngeal cancer: A retrospective study

**DOI:** 10.1097/MD.0000000000050411

**Published:** 2026-08-28

**Authors:** Cheng Jin, Yi Zhang

**Affiliations:** a Department of Otorhinolaryngology, Pujiang Hospital, Shanghai, China; b Department of Otorhinolaryngology, Renji Hospital Affiliated to Shanghai Jiao Tong University School of Medicine, Shanghai, China.

**Keywords:** dysphagia, Global Leadership Initiative on Malnutrition, laryngeal cancer, postoperative malnutrition, risk factors, total laryngectomy

## Abstract

Postoperative malnutrition is a frequent yet variably defined complication after laryngeal cancer surgery and may compromise functional rehabilitation and subsequent oncologic management. This retrospective cohort study consecutively included adult patients (≥ 18 years) with histopathologically confirmed primary laryngeal cancer who underwent curative-intent surgery at our institution between July 1, 2021 and July 31, 2025. The primary endpoint was postoperative malnutrition within 3 months, diagnosed using the Global Leadership Initiative on Malnutrition criteria. Among 215 eligible patients, 56 developed postoperative malnutrition, yielding an incidence of 26.0% (56/215; 95% confidence interval, 20.6–32.3%). The median time to first Global Leadership Initiative on Malnutrition-defined malnutrition was 24 days (interquartile range, 14–41), with 60.7% of events occurring within 30 days postoperatively. Patients who developed malnutrition were older and had lower preoperative body mass index and less favorable tumor and functional profiles, including higher rates of advanced tumor–node–metastasis stage and preoperative dysphagia. Univariable and multivariable logistic regression identified independent associations with postoperative malnutrition, including lower body mass index and serum albumin, higher C-reactive protein, advanced tumor–node–metastasis stage, preoperative dysphagia, total laryngectomy, and longer operative duration. These findings support risk-stratified perioperative nutritional surveillance and early multidisciplinary interventions in laryngeal cancer surgery.

## 1. Introduction

Laryngeal cancer remains a major subtype of head and neck cancer (HNC) that frequently requires surgery as a definitive treatment, including partial or total laryngectomy with or without neck dissection and reconstructive procedures. In this setting, postoperative recovery is commonly complicated by impaired oral intake due to anatomical and functional alterations of the upper aerodigestive tract, pain, airway diversion, and treatment-related inflammation. Nutritional deterioration is, therefore, a recurring clinical concern, with implications for functional rehabilitation, tolerance of subsequent oncologic therapies, and overall outcomes. Contemporary observational data in advanced HNC indicate that malnutrition is common and is associated with worse health-related quality of life, underscoring the clinical relevance of nutrition-focused supportive care across the treatment trajectory.^[1,2]^ A central challenge in perioperative nutrition research is the heterogeneity of malnutrition definitions and assessment approaches. The Global Leadership Initiative on Malnutrition (GLIM) criteria provide a consensus framework that integrates phenotypic and etiologic domains and has been increasingly applied in oncology and HNC populations. In a recent diagnostic meta-analysis in adults with cancer, GLIM demonstrated moderate diagnostic performance against the Patient-Generated Subjective Global Assessment, and GLIM-defined malnutrition was associated with adverse survival and complication endpoints, supporting its use as a clinically meaningful construct.^[3,4]^ Beyond diagnosis, GLIM-defined malnutrition and nutrition-impact symptoms have been linked to longer-term functional recovery metrics in HNC, suggesting that early nutritional deficits may have downstream consequences extending beyond the immediate postoperative period.^[5]^

Multiple mechanisms plausibly contribute to postoperative malnutrition following laryngeal cancer surgery. First, nutrition-impact symptoms (particularly dysphagia) can directly constrain intake and delay the reestablishment of adequate oral feeding. Second, systemic inflammation and surgical stress can promote catabolism and anorexia, potentially amplifying preexisting nutritional vulnerability. Third, procedure-related complexity may prolong reliance on tube feeding and increase intensive care utilization, thereby influencing nutritional trajectories. Recent perioperative reviews in HNC reconstruction emphasize the potential value of integrating nutritional biomarkers and metabolic risk profiling into perioperative pathways.^[6]^ Prehabilitation literature highlights the rationale for multidisciplinary programs incorporating nutrition and functional optimization in HNC, although implementation remains variable and evidence gaps persist.^[7,8]^ Within laryngectomy cohorts, biochemical indices reflecting nutritional reserve have been associated with postoperative wound-related complications, suggesting clinical utility for routine nutritional laboratory assessment in high-risk surgical candidates.^[9]^ Emerging translational studies in laryngeal cancer further support links between inflammatory regulation and nutritional phenotypes, reinforcing the biologic plausibility that inflammation and nutritional status are interrelated in this disease context.^[10]^ In addition, randomized evidence evaluating perioperative immunonutrition in HNC surgery continues to develop, reflecting ongoing efforts to translate nutritional risk recognition into actionable interventions. However, despite increasing attention to perioperative nutrition in HNC, data remain limited regarding GLIM-defined postoperative malnutrition within the early post-discharge period and its clinical correlates specifically among surgically treated laryngeal cancer patients.^[11]^

Accordingly, the present retrospective study evaluated the incidence of postoperative malnutrition within 3 months after laryngeal cancer surgery using GLIM criteria and examined demographic, tumor-related, functional, perioperative, and laboratory factors associated with this endpoint to inform risk-stratified perioperative nutritional surveillance and supportive care.

## 2. Methods

### 2.1. Study design

This study was approved by the Ethics Committee of Renji Hospital Affiliated with Shanghai Jiao Tong University School of Medicine. This retrospective cohort study consecutively screened adult patients (≥ 18 years) with histopathologically confirmed primary laryngeal cancer who underwent surgical treatment at our institution between July 1, 2021 and July 31, 2025. The sole outcome-assessment window was the first 3 months (90 days) after surgery. Nutritional information recorded during the index hospitalization and at postoperative follow-up visits was used as repeated source data within this single 90-day window; hospitalization and postoperative day 30 were not treated as separate outcome windows. Patients meeting the diagnostic criteria for postoperative malnutrition at any time from surgery through postoperative day 90 were classified as the malnutrition group, whereas those who did not meet the criteria throughout this period constituted the control group. Eligibility criteria were defined a priori: inclusion criteria comprised: definitive diagnosis of laryngeal cancer, receipt of primary curative-intent laryngeal surgery at our center, and availability of complete perioperative records necessary for nutritional classification and risk factor ascertainment (including preoperative baseline data and follow-up information through the 3-month assessment window). Exclusion criteria were: age < 18 years; recurrent laryngeal cancer, distant metastasis at baseline, or history of other active malignancies; preexisting malnutrition prior to surgery according to the same criteria applied postoperatively, or inability to determine baseline nutritional status due to missing data; receipt of preoperative anticancer therapy (neoadjuvant chemotherapy and/or radiotherapy); major comorbid conditions independently associated with malnutrition or acute severe infection at admission; perioperative death or loss to follow-up before completion of the 90-day nutritional assessment; and incomplete key variables required for multivariable modeling. The study was conducted in accordance with the Declaration of Helsinki and was approved by the institutional medical ethics committee. Written informed consent for the use of clinical data was obtained from all participants; where applicable in retrospective research, informed consent was obtained at admission or waived by the ethics committee in accordance with local regulations and institutional policy.

### 2.2. Outcome definition and group allocation

The primary outcome was postoperative malnutrition occurring within 3 months (postoperative days 0–90) after surgery. Postoperative malnutrition was diagnosed according to the GLIM framework, requiring at least 1 phenotypic criterion together with at least 1 etiologic criterion. For phenotypic criteria, non-volitional weight loss was defined as > 5% within the preceding 6 months or > 10% beyond 6 months, and low body mass index (BMI) was defined using the Asian GLIM cutoffs of < 18.5 kg/m^2^ for patients aged < 70 years and < 20.0 kg/m^2^ for those aged ≥ 70 years. Routine computed-tomography, dual-energy X-ray absorptiometry, bioelectrical-impedance, or ultrasound-based muscle-mass measurements were not consistently available in the retrospective records; therefore, reduced muscle mass was not used as an independent phenotypic criterion in the primary classification. For etiologic criteria, reduced food intake was defined as intake ≤ 50% of estimated energy requirements for > 1 week or any clinically meaningful reduction in intake persisting for > 2 weeks, based on dietitian, nursing, and physician documentation. Reduced assimilation was considered when persistent gastrointestinal dysfunction or a documented malabsorptive condition was present. Disease burden/inflammation was considered present in the context of active laryngeal cancer and the postoperative inflammatory state, with C-reactive protein (CRP) used as a supportive quantitative indicator rather than as the sole basis for classification. Nutritional status was evaluated from routinely collected anthropometric and clinical data during hospitalization and follow-up through postoperative day 90. For each patient, the earliest date on which the required phenotypic and etiologic criteria were simultaneously met was recorded as the event date.

Patients were categorized into 2 groups based on the primary outcome. The malnutrition group (case group) comprised patients who met the GLIM diagnostic criteria for malnutrition within 3 months after surgery. The control group included patients who did not meet GLIM criteria for malnutrition throughout the 3-month postoperative assessment window. Outcome classification was independently performed by 2 trained investigators who were blinded to each other’s assessments and followed a standardized adjudication protocol. Any discrepancies in GLIM criterion interpretation or final group assignment were resolved through rereview of the source records and consensus discussion. If disagreement persisted, a third senior investigator served as an adjudicator and made the final determination.

### 2.3. Candidate risk factors and variable definitions

Prespecified candidate risk factors were extracted from the electronic medical record using a standardized data collection form. Baseline variables were defined using the preoperative assessment closest to surgery, and laboratory indices were defined as the last available preoperative measurements. Baseline demographic variables included age (years), sex, BMI (kg/m^2^), smoking history, and alcohol use as recorded in the admission and preoperative evaluation records. Tumor-related variables included tumor–node–metastasis (TNM) stage, primary tumor subsite (glottic/supraglottic/subglottic), and histologic differentiation grade based on pathology and staging documentation. Preoperative laryngopharyngeal functional status was collected from otolaryngology assessments, including dysphagia and voice function impairment.

Perioperative treatment and surgery-related variables comprised surgical procedure type (partial vs total laryngectomy), operative duration, estimated blood loss, neck dissection, tracheostomy, and reconstruction method as documented in operative and anesthesia records. Postoperative nutritional support was categorized by modality (enteral and/or parenteral) and feeding route (nasogastric tube or gastrostomy). Intensive care unit (ICU) admission and postoperative mechanical ventilation were also recorded when applicable. Comorbidities and perioperative risk assessment included American Society of Anesthesiologists (ASA) physical status (if available), diabetes mellitus, hypertension, and chronic obstructive pulmonary disease. Preoperative anemia, inflammatory indices, and hepatic/renal function parameters were collected from the most recent preoperative laboratory tests.

### 2.4. Statistical analysis

All statistical analyses were performed using IBM SPSS Statistics, version 26.0 (IBM Corp.) and R software, version 4.3.2 (R Foundation for Statistical Computing). Continuous variables were assessed for distributional characteristics and summarized as mean ± standard deviation when approximately normally distributed; otherwise, they were presented as median (interquartile range). Categorical variables were reported as number (percentage). Between-group comparisons were conducted using the independent-samples *t*-test for continuous variables and the Pearson *χ*^2^ test for categorical variables; Fisher exact test was applied when expected cell counts were small. Univariable logistic regression was used to evaluate associations between each candidate factor and postoperative malnutrition within 3 months, with results reported as odds ratios (ORs) and 95% confidence intervals (CIs). Confounding control was planned around variables measured before the occurrence of the nutritional outcome. Age was retained as a basic demographic confounder, and baseline nutritional reserve (BMI and albumin), inflammatory burden (CRP), disease severity (TNM stage), functional impairment (preoperative dysphagia), and surgical burden (laryngectomy type and operative duration) were included because of their clinical relevance and/or univariable association. Highly overlapping markers of surgical complexity were not entered simultaneously, and postoperative variables that could represent consequences of early nutritional deterioration (such as ICU admission, mechanical ventilation, feeding route, and intensified nutritional support) were excluded from the final model to reduce overadjustment, reverse causation, and indication bias. The number of covariates was constrained in view of the 56 outcome events. Multivariable logistic regression results were reported as adjusted ORs (aORs), 95% CIs, and 2-sided *P* values. Complete-case analysis was applied for regression modeling, excluding records with missing covariate data. Because residual confounding by unmeasured variables remained possible, the adjusted estimates were interpreted as independent associations rather than causal effects. Statistical significance was defined as a 2-sided *P* value < .05.

## 3. Results

### 3.1. Primary outcome and group allocation

Among 215 eligible patients who underwent primary surgery for laryngeal cancer, 56 patients developed postoperative malnutrition within 3 months according to the GLIM criteria, yielding an overall incidence of 26.0% (56/215; 95% CI, 20.6–32.3%). The median time to the first GLIM-defined malnutrition event was 24 days (interquartile range, 14–41). In the malnutrition cohort, events were predominantly observed early after surgery: 60.7% (34/56) occurred within the first 30 postoperative days, including 32.1% (18/56) within ≤ 14 days and 28.6% (16/56) during days 15 to 30. The remaining events occurred between days 31 to 60 (25.0%, 14/56) and days 61 to 90 (14.3%, 8/56). Based on endpoint status, 56 patients constituted the malnutrition group, and 159 patients constituted the non-malnutrition control group.

### 3.2. Baseline demographics, lifestyle, tumor, and functional characteristics

Patients who developed postoperative malnutrition were older than those in the control group (63.2 ± 7.8 vs 60.1 ± 8.5 years; *t* = 2.50, *P* = .014) and had a lower preoperative BMI (21.3 ± 2.6 vs 23.0 ± 2.8 kg/m^2^; *t* = −4.12, *P* < .001), whereas the sex distribution was similar between groups (*P* = .974). Smoking status differed significantly (*χ*^2^ = 6.49, *P* = .039), with a higher proportion of current smokers in the malnutrition group; by contrast, alcohol-use categories did not differ between groups (*χ*^2^ = 2.76, *P* = .252). Regarding oncologic features, the malnutrition group more frequently presented with advanced TNM stage (III–IV) (73.2% vs 49.7%; *χ*^2^ = 8.37, *P* = .004), showed a different distribution of primary subsites (*χ*^2^ = 10.24, *P* = .006), and had a higher prevalence of poor differentiation (*χ*^2^ = 8.67, *P* = .013). Preoperative dysphagia and voice impairment were also more common among patients who subsequently developed malnutrition (58.9% vs 33.3%; *χ*^2^ = 10.26, *P* = .001; and 78.6% vs 57.9%; *χ*^2^ = 6.78, *P* = .009, respectively) (Table 1).

**Table 1:** Baseline demographics, lifestyle, tumor, and preoperative functional characteristics by postoperative malnutrition status.

Variable	Malnutrition (n = 56)	Control (n = 159)	Test statistic	*P* value
Demographics and lifestyle				
Age, yrs (mean ± SD)	63.2 ± 7.8	60.1 ± 8.5	*t* = 2.50	.014
Male sex, n (%)	45 (80.4)	130 (81.8)	*χ*^2^ = 0.00	.974
BMI, kg/m^2^ (mean ± SD)	21.3 ± 2.6	23.0 ± 2.8	*t* = −4.12	< .001
Smoking status (current/former/never), n (%)	24 (42.9)/ 14 (25.0)/ 18 (32.1)	42 (26.4)/ 38 (23.9)/ 79 (49.7)	*χ*^2^ = 6.49	.039
Alcohol use (current/former/never), n (%)	22 (39.3)/ 10 (17.9)/ 24 (42.9)	44 (27.7)/ 30 (18.9)/ 85 (53.5)	*χ*^2^ = 2.76	.252
Tumor and functional characteristics				
TNM stage (I–II/ III–IV), n (%)	15 (26.8)/ 41 (73.2)	80 (50.3)/ 79 (49.7)	*χ*^2^ = 8.37	.004
Primary subsite (glottic/supraglottic/subglottic), n (%)	20 (35.7)/ 30 (53.6)/ 6 (10.7)	96 (60.4)/ 51 (32.1)/ 12 (7.5)	*χ*^2^ = 10.24	.006
Differentiation (well/moderate/poor), n (%)	10 (17.9)/ 27 (48.2)/ 19 (33.9)	48 (30.2)/ 85 (53.5)/ 26 (16.4)	*χ*^2^ = 8.67	.013
Preoperative dysphagia, n (%)	33 (58.9)	53 (33.3)	*χ*^2^ = 10.26	.001
Preoperative voice impairment, n (%)	44 (78.6)	92 (57.9)	*χ*^2^ = 6.78	.009

BMI = body mass index, n = number of patients, SD = standard deviation, TNM = tumor–node–metastasis.

### 3.3. Perioperative factors, comorbidities, and preoperative laboratory indices

Perioperative profiles suggested greater surgical complexity and higher postoperative care intensity in the malnutrition group, including higher rates of total laryngectomy (*χ*^2^ = 7.05, *P* = .008), longer operative duration (215 ± 55 vs 185 ± 50 minutes; *t* = 3.59, *P* < .001), and greater estimated blood loss (320 ± 180 vs 240 ± 150 mL; *t* = 2.98, *P* = .004). Neck dissection patterns differed between groups (*χ*^2^ = 10.04, *P* = .007), and tracheostomy and flap reconstruction were more frequent in the malnutrition group (*χ*^2^ = 8.48, *P* = .004; *χ*^2^ = 8.91, *P* = .003). Postoperative nutritional support modalities and feeding routes also differed (*χ*^2^ = 12.33, *P* = .002; *χ*^2^ = 12.61, *P* = .002), and ICU admission and mechanical ventilation occurred more often among patients with postoperative malnutrition (*χ*^2^ = 9.59, *P* = .002; *χ*^2^ = 8.75, *P* = .003). With respect to comorbidities and perioperative risk, ASA class III to IV was more prevalent in the malnutrition group (*χ*^2^ = 4.45, *P* = .035), whereas diabetes mellitus, hypertension, and chronic obstructive pulmonary disease did not differ significantly (all *P* > .05). For preoperative laboratory indices, the malnutrition group had lower hemoglobin and albumin levels and higher CRP concentrations (all *P* ≤ .004), while serum creatinine levels were comparable between groups (*t* = 1.47, *P* = .145) (Table 2).

**Table 2:** Perioperative factors, comorbidities, perioperative risk, and preoperative laboratory indices by postoperative malnutrition status.

Variable	Malnutrition (n = 56)	Control (n = 159)	Test statistic	*P* value
Perioperative and surgery-related factors				
Procedure (partial/total laryngectomy), n (%)	22 (39.3)/ 34 (60.7)	97 (61.0)/ 62 (39.0)	*χ*^2^ = 7.05	.008
Operative duration, min (mean ± SD)	215 ± 55	185 ± 50	*t* = 3.59	< .001
Estimated blood loss, mL (mean ± SD)	320 ± 180	240 ± 150	*t* = 2.98	.004
Neck dissection (none/unilateral/bilateral), n (%)	6 (10.7)/ 24 (42.9)/ 26 (46.4)	40 (25.2)/ 78 (49.1)/ 41 (25.8)	*χ*^2^ = 10.04	.007
Tracheostomy, n (%)	38 (67.9)	70 (44.0)	*χ*^2^ = 8.48	.004
Reconstruction (flap), n (%)	21 (37.5)	27 (17.0)	*χ*^2^ = 8.91	.003
Postoperative nutrition support (EN only/PN only/EN + PN), n (%)	20 (35.7)/ 14 (25.0)/ 22 (39.3)	98 (61.6)/ 18 (11.3)/ 43 (27.0)	*χ*^2^ = 12.33	.002
Feeding route (NGT/gastrostomy/oral), n (%)	34 (60.7)/ 15 (26.8)/ 7 (12.5)	84 (52.8)/ 20 (12.6)/ 55 (34.6)	*χ*^2^ = 12.61	.002
ICU admission, n (%)	18 (32.1)	20 (12.6)	*χ*^2^ = 9.59	.002
Mechanical ventilation, n (%)	12 (21.4)	10 (6.3)	*χ*^2^ = 8.75	.003
Comorbidities, perioperative risk, and preoperative labs				
ASA class III–IV, n (%)	29 (51.8)	55 (34.6)	*χ*^2^ = 4.45	.035
Diabetes mellitus, n (%)	11 (19.6)	18 (11.3)	*χ*^2^ = 1.80	.180
Hypertension, n (%)	26 (46.4)	62 (39.0)	*χ*^2^ = 0.66	.415
COPD, n (%)	9 (16.1)	12 (7.5)	*χ*^2^ = 2.52	.113
Hemoglobin, g/L (mean ± SD)	118 ± 16	126 ± 15	*t* = −3.27	.002
Albumin, g/L (mean ± SD)	36.1 ± 4.2	39.0 ± 3.9	*t* = −4.53	< .001
CRP, mg/L (mean ± SD)	12.5 ± 8.0	9.0 ± 6.5	*t* = 2.95	.004
Creatinine, µmol/L (mean ± SD)	78 ± 18	74 ± 16	*t* = 1.47	.145

ASA = American Society of Anesthesiologists, COPD = chronic obstructive pulmonary disease, CRP = C-reactive protein, EN = enteral nutrition, ICU = intensive care unit, n = number of patients, NGT = nasogastric tube, PN = parenteral nutrition, SD = standard deviation.

### 3.4. Univariable analysis of factors associated with postoperative malnutrition

In univariable logistic regression, older age and lower BMI were associated with higher odds of postoperative malnutrition (age per year: OR 1.05, *P* = .015; BMI per kg/m^2^: OR 0.78, *P* < .001). Current smoking was associated with increased odds compared with never smoking (OR 2.51, *P* = .007). Tumor- and function-related variables associated with malnutrition included advanced TNM stage, supraglottic subsite, poor differentiation, preoperative dysphagia, and voice impairment (all *P* ≤ .006). Several perioperative indicators of surgical complexity and postoperative care intensity were also associated with malnutrition, including total laryngectomy, longer operative duration, greater blood loss, bilateral neck dissection, tracheostomy, flap reconstruction, intensified nutrition support (parenteral nutrition only or enteral nutrition + parenteral nutrition vs enteral nutrition only), non-oral feeding routes (nasogastric tube or gastrostomy vs. oral), ICU admission, and mechanical ventilation (all *P* ≤ .008). Among comorbidity and laboratory indices, ASA class III to IV increased the odds of malnutrition (OR 2.03, *P* = .019), whereas higher hemoglobin and albumin were protective, and higher CRP was associated with increased odds (all *P* ≤ .003); creatinine was not significantly associated (*P* = .200) (Table 3).

**Table 3:** Univariable logistic regression for postoperative malnutrition within 3 months.

Variable	Reference	OR (95% CI)	*P* value
Demographics and lifestyle			
Age (per 1-year increase)	-	1.05 (1.01–1.09)	.015
Male sex	Female	0.92 (0.45–1.88)	.816
BMI (per 1 kg/m^2^ increase)	-	0.78 (0.69–0.88)	< .001
Smoking: current	Never	2.51 (1.28–4.92)	.007
Smoking: former	Never	1.62 (0.78–3.37)	.198
Alcohol: current	Never	1.77 (0.95–3.30)	.072
Alcohol: former	Never	1.18 (0.53–2.63)	.690
Tumor and functional characteristics			
Advanced TNM stage (III–IV)	I–II	2.77 (1.44–5.33)	.002
Subsite: supraglottic	Glottic	2.82 (1.49–5.33)	.001
Subsite: subglottic	Glottic	2.40 (0.86–6.72)	.094
Differentiation: moderate	Well	1.53 (0.66–3.55)	.320
Differentiation: poor	Well	3.50 (1.47–8.36)	.005
Preoperative dysphagia	No	2.87 (1.56–5.28)	.001
Preoperative voice impairment	No	2.65 (1.32–5.32)	.006
Perioperative and surgery-related factors			
Total laryngectomy	Partial	2.42 (1.33–4.41)	.004
Operative duration (per 10 min)	-	1.11 (1.06–1.17)	< .001
Estimated blood loss (per 100 mL)	-	1.28 (1.10–1.50)	.002
Neck dissection: unilateral	None	2.05 (0.78–5.38)	.145
Neck dissection: bilateral	None	4.23 (1.60–11.16)	.003
Tracheostomy	No	2.69 (1.45–4.99)	.002
Flap reconstruction	Primary closure	2.92 (1.48–5.77)	.002
Nutrition support: PN only	EN only	3.81 (1.63–8.90)	.002
Nutrition support: EN + PN	EN only	2.50 (1.33–4.70)	.004
Feeding route: NGT	Oral	3.18 (1.36–7.45)	.008
Feeding route: gastrostomy	Oral	5.89 (2.40–14.45)	< .001
ICU admission	No	3.26 (1.62–6.57)	.001
Mechanical ventilation	No	4.05 (1.69–9.73)	.002
Comorbidities and preoperative labs			
ASA class III–IV	I–II	2.03 (1.12–3.68)	.019
Diabetes mellitus	No	1.92 (0.86–4.28)	.110
Hypertension	No	1.35 (0.75–2.41)	.322
COPD	No	2.34 (0.92–5.91)	.074
Hemoglobin (per 10 g/L)	-	0.64 (0.50–0.82)	< .001
Albumin (per 1 g/L)	-	0.83 (0.77–0.90)	< .001
CRP (per 5 mg/L)	-	1.22 (1.07–1.40)	.003
Creatinine (per 10 µmol/L)	-	1.10 (0.95–1.28)	.200

ASA = American Society of Anesthesiologists, BMI = body mass index, CI = confidence interval, COPD = chronic obstructive pulmonary disease, CRP = C-reactive protein, EN = enteral nutrition, ICU = intensive care unit, NGT = nasogastric tube, OR = odds ratio, PN = parenteral nutrition, TNM = tumor–node–metastasis.

### 3.5. Multivariable logistic regression analysis

In the multivariable logistic regression model, lower preoperative BMI and lower preoperative albumin were independently associated with higher odds of postoperative malnutrition (BMI: aOR 0.82 per 1 kg/m^2^, *P* < .001; albumin: aOR 0.88 per 1 g/L, *P* < .001). Indicators of greater disease burden and perioperative stress were also independently associated with malnutrition, including advanced TNM stage (III–IV vs. I–II; aOR 2.19, *P* = .030), preoperative dysphagia (aOR 2.10, *P* = .034), total laryngectomy (aOR 1.98, *P* = .044), longer operative duration (aOR 1.07 per 10 minutes, *P* = .006), and higher preoperative CRP (aOR 1.17 per 5 mg/L, *P* = .028). Age did not remain statistically significant after adjustment (*P* = .112) (Table 4). After adjustment for covariates, advanced TNM stage, preoperative dysphagia, and more extensive surgery (total laryngectomy) with longer operative duration were directionally associated with an increased risk of postoperative malnutrition, consistent with a higher perioperative disease and treatment burden. Conversely, higher BMI and albumin were associated with reduced odds of malnutrition, suggesting that better preoperative nutritional reserve was linked to a lower postoperative malnutrition risk. Higher CRP was associated with increased odds, indicating that a greater preoperative inflammatory burden correlated with postoperative malnutrition occurrence within 3 months.

**Table 4:** Multivariable logistic regression for postoperative malnutrition within 3 months.

Variable	aOR (95% CI)	*P* value
Age (per 1-year increase)	1.03 (0.99–1.08)	.112
BMI (per 1 kg/m^2^ increase)	0.82 (0.74–0.92)	< .001
Advanced TNM stage (III–IV vs I–II)	2.19 (1.08–4.45)	.030
Preoperative dysphagia (yes vs no)	2.10 (1.06–4.16)	.034
Total laryngectomy (vs partial laryngectomy)	1.98 (1.02–3.87)	.044
Operative duration (per 10-min increase)	1.07 (1.02–1.12)	.006
Albumin (per 1 g/L increase)	0.88 (0.82–0.95)	< .001
CRP (per 5 mg/L increase)	1.17 (1.02–1.35)	.028

aOR = adjusted odds ratio, BMI = body mass index, CI = confidence interval, CRP = C-reactive protein, GLIM = Global Leadership Initiative on Malnutrition, TNM = tumor–node–metastasis.

## 4. Discussion

In this retrospective cohort of patients undergoing primary surgical treatment for laryngeal cancer, postoperative malnutrition within 3 months, defined using the GLIM framework, occurred in approximately 1-quarter of patients (26.0%). This incidence is clinically meaningful and is broadly consistent with contemporary evidence indicating that malnutrition and nutritional risk are common around major HNC surgery and are associated with adverse postoperative outcomes.^[12]^ Collectively, the present findings support the need for structured perioperative nutritional risk stratification in laryngeal cancer surgery and identify a set of variables that may inform targeted prevention strategies.

In multivariable analysis, lower preoperative BMI and lower preoperative albumin were independently associated with higher odds of postoperative malnutrition. These associations are directionally consistent with the concept that reduced nutritional reserve and impaired protein status increase vulnerability to postoperative catabolism and reduced intake. While albumin is not a nutrition-specific biomarker and is influenced by inflammation and fluid status, its association with postoperative complications and impaired healing has been repeatedly observed in surgical oncology, including laryngectomy-related complications.^[13]^ In this context, the persistence of albumin as an independent factor after adjustment (alongside CRP) suggests that both baseline physiologic reserve and inflammatory burden contribute to postoperative nutritional deterioration. Higher preoperative CRP also remained independently associated with postoperative malnutrition. CRP is a nonspecific marker of systemic inflammation and may reflect tumor-related inflammation, comorbidity-associated inflammatory states, or subclinical infection. Inflammatory signaling is mechanistically linked to anorexia, altered macronutrient metabolism, and muscle catabolism; therefore, elevated CRP may identify patients in whom postoperative nutritional decline is more likely despite standard perioperative pathways. Recent literature in laryngectomy populations has similarly highlighted the clinical relevance of CRP (often alongside albumin and hemoglobin) in relation to postoperative wound complications.^[14,15]^ Although the present analysis does not establish causality, the observed association supports incorporating inflammatory markers into preoperative nutritional risk assessment, particularly when GLIM criteria are used (which explicitly include disease burden/inflammation as an etiologic domain).

Advanced TNM stage was independently associated with postoperative malnutrition. This finding is plausible given that higher-stage disease is commonly accompanied by greater tumor burden, more pronounced symptomatology (including odynophagia and dysphagia), and increased likelihood of extensive surgery and adjunctive treatments. In addition, advanced disease may be associated with longer prediagnostic symptom duration and baseline weight loss, thereby reducing preoperative reserve. Contemporary surgical cohorts in HNC have reported that malnutrition is common and clinically consequential, reinforcing the importance of systematic preoperative screening in this population.^[16]^ The present results extend this concept specifically to laryngeal cancer surgery when postoperative malnutrition is defined using a standardized consensus framework. Preoperative dysphagia remained an independent correlate of postoperative malnutrition. Dysphagia is a central “nutrition-impact symptom” in HNC that can directly limit oral intake and contribute to progressive weight loss, sarcopenia, and reduced functional capacity.^[17,18]^ In laryngeal cancer, dysphagia may reflect local tumor effects, baseline airway–digestive tract compromise, and reduced compensatory swallowing function, which can be exacerbated postoperatively by pain, edema, and reconstruction-related changes. Evidence increasingly supports early swallow-focused interventions (“prehabilitation”) and proactive dysphagia management to preserve swallowing function and mitigate downstream nutritional consequences.^[19]^ While the current study did not evaluate specific swallowing therapy protocols, the independent association between dysphagia and postoperative malnutrition suggests that integrating structured dysphagia screening and early swallow rehabilitation into perioperative pathways may be clinically relevant in this population. Total laryngectomy and longer operative duration were also independently associated with postoperative malnutrition. These factors likely capture procedure extent and surgical stress, which can influence postoperative inflammatory response, recovery trajectory, and the duration of impaired oral intake. Open laryngeal procedures and complex reconstructions often necessitate prolonged reliance on enteral feeding and are associated with higher complication risk, which can further delay rehabilitation and nutritional recovery. Recent laryngectomy-focused evidence has emphasized the relationship between preoperative nutritional status and postoperative complications, underscoring a bidirectional relationship in which operative complexity and nutritional vulnerability may amplify one another.^[20]^ These observations support multidisciplinary perioperative planning that anticipates feeding needs, optimizes analgesia and mobilization, and enables early transition to adequate enteral intake when feasible.

The present findings can be translated into a practical, risk-stratified pathway. Patients with low BMI or albumin, elevated CRP, advanced-stage disease, preoperative dysphagia, or an anticipated total laryngectomy/long operation could be flagged at the preoperative visit for dietitian assessment and individualized energy–protein planning. Dysphagia screening and speech-language pathology input should occur before surgery, with early postoperative reassessment to determine the safest oral texture and the need for enteral support. During admission, actual intake, weight, inflammatory status, and feeding tolerance should be reviewed repeatedly; failure to achieve approximately 60% of estimated requirements despite oral strategies should prompt timely escalation of oral nutritional supplements or enteral feeding according to clinical judgment. After discharge, focused nutritional review at approximately 2, 4, 8, and 12 weeks would cover the period in which events accumulated in this cohort and permit rapid correction of declining intake or weight. These recommendations represent a proposed application of the observed risk associations and should be prospectively tested rather than interpreted as intervention effects established by the present retrospective study. Compared with previous reports that frequently combined heterogeneous HNC sites, emphasized preoperative nutritional status or short-term surgical complications, or evaluated patients receiving nonsurgical treatment, this study focused specifically on surgically treated laryngeal cancer and used a uniform GLIM-defined 90-day postoperative endpoint. The simultaneous consideration of nutritional reserve, CRP, dysphagia, tumor stage, and operative complexity also provides a more integrated view of biological, functional, and treatment-related vulnerability.

Several methodological features strengthen interpretability. Postoperative malnutrition was defined using GLIM criteria and evaluated within 1 prespecified 3-month (90-day) window, providing clinically meaningful temporal alignment with the period of greatest postoperative nutritional vulnerability. In addition, endpoint adjudication used a structured process with independent review, which may reduce misclassification. However, limitations should be recognized. The retrospective, single-center design limits causal inference and may restrict generalizability. Residual confounding is possible despite the clinically structured multivariable adjustment, particularly for unmeasured factors such as baseline sarcopenia, detailed dietary intake, socioeconomic determinants, standardized symptom severity, postoperative complications, and rehabilitation adherence. GLIM implementation was constrained by the absence of consistently available objective muscle-mass measurements; consequently, the primary phenotypic classification relied on documented weight loss and age-specific Asian BMI thresholds. This approach may underestimate malnutrition in patients with preserved or elevated BMI but low muscle mass, including sarcopenic obesity. The retrospective estimation of reduced intake from clinical documentation may also be less precise than prospective calorie counts. In addition, perioperative nutrition-support variables can be affected by indication bias (i.e., clinicians may intensify nutrition support in response to early nutritional decline), and thus associations observed in univariable analyses should not be interpreted as causal effects. Finally, complete-case analysis may introduce selection bias if missingness was not completely at random.

## 5. Conclusion

In summary, GLIM-defined postoperative malnutrition within 3 months after laryngeal cancer surgery was common. Lower preoperative BMI and albumin levels, higher preoperative CRP, advanced TNM stage, preoperative dysphagia, total laryngectomy, and longer operative duration were independently associated with higher odds of postoperative malnutrition. These routinely available clinical factors may help identify patients who require intensified perioperative nutritional surveillance, early dietetic assessment, and proactive dysphagia management. Given the retrospective observational design and the possibility of residual confounding, these findings should be interpreted as associations rather than causal relationships and require validation in prospective multicenter studies.

## Author contributions

**Conceptualization:** Cheng Jin, Yi Zhang.

**Data curation:** Cheng Jin, Yi Zhang.

**Formal analysis:** Cheng Jin, Yi Zhang.

**Funding acquisition:** Yi Zhang.

**Investigation:** Yi Zhang.

**Writing** – **original draft:** Yi Zhang.

**Writing** – **review & editing:** Yi Zhang.

## References

[1] Wallmander C Bosaeus I Silander E. Malnutrition in patients with advanced head and neck cancer: exploring the Global Leadership Initiative on Malnutrition (GLIM) criteria, energy balance and health-related quality of life. Clin Nutr ESPEN. 2025;66:332–42. 39892786 10.1016/j.clnesp.2025.01.049

[2] Berg M Hansson C Silander E. A randomized study comparing the nutritional effects of radiotherapy with cetuximab versus cisplatin in patients with advanced head and neck cancer. Head Neck. 2024;46:760–71. 38192119 10.1002/hed.27619

[3] Zhou J Yang S Liu T Sun Y Li S. Diagnostic performance of GLIM and PG-SGA for malnutrition assessment in adult cancer patients: a systematic review and meta-analysis. BMC Cancer. 2025;25:765. 40269782 10.1186/s12885-025-13809-6 PMC12020302

[4] Wu T Zhou M Xu K. GLIM achieves best diagnostic performance in non-cancer patients with low BMI: a hierarchical Bayesian latent-class meta-analysis. Nutr Rev. 2025;83:e877–91. 39013202 10.1093/nutrit/nuae096

[5] Einarsson S Bokström A Laurell G Tiblom Ehrsson Y. Mapping the impact of malnutrition as defined by the Global Leadership Initiative on Malnutrition and nutrition impact symptoms on the possibility of returning to work after treatment for head and neck cancer. Support Care Cancer. 2023;32:55. 38133825 10.1007/s00520-023-08252-x PMC10746764

[6] Jaxa-Kwiatkowski A Jaxa-Kwiatkowski M Jaxa-Kwiatkowska K Gerber H Kubiak M Łysenko L. Perioperative nutritional and metabolic factors affecting surgical outcomes in head and neck cancer free flap reconstruction: a comprehensive review. J Clin Med. 2025;14:3679. 40507441 10.3390/jcm14113679 PMC12155434

[7] Demurtas S Cena H Benazzo M. Head and Neck Cancer (HNC) prehabilitation: advantages and limitations. J Clin Med. 2024;13:6176. 39458125 10.3390/jcm13206176 PMC11509296

[8] Baker K Roe J Brady G Cruickshank S. Prehabilitation and rehabilitation guidelines for allied healthcare professionals working in head and neck cancer: a narrative review. Disabil Rehabil. 2025;48:3943–58. 41268643 10.1080/09638288.2025.2587005

[9] Tang A Dang S Lao I. Association of prealbumin with complications after total laryngectomy with free flap reconstruction. Am J Otolaryngol. 2024;45:104451. 39137698 10.1016/j.amjoto.2024.104451

[10] Mazurek M Brzozowska A Maziarz M Małecka-Massalska T Powrózek T. The relationship between miR-5682 and nutritional status of radiotherapy-treated male laryngeal cancer patients. Genes (Basel). 2024;15:556. 38790185 10.3390/genes15050556 PMC11120884

[11] Chen X Beilman B Gibbs HD. Nutrition in head and neck cancer care: a roadmap and call for research. Lancet Oncol. 2025;26:e300–10. 40449504 10.1016/S1470-2045(25)00087-7 PMC12915487

[12] Reed WT Jiang R Ohnuma T. Malnutrition and adverse outcomes after surgery for head and neck cancer. JAMA Otolaryngol Head Neck Surg. 2024;150:14–21. 37883116 10.1001/jamaoto.2023.3486 PMC10603580

[13] Qi AC Peacock K Luke AA Barker A Olsen MA Joynt Maddox KE. Associations between social risk factors and surgical site infections after colectomy and abdominal hysterectomy. JAMA Netw Open. 2019;2:e1912339. 31577353 10.1001/jamanetworkopen.2019.12339 PMC6777422

[14] Thompson ER Abdel-Azim N Yan K. Nutritional status and outcomes following open laryngeal surgery for laryngeal cancer: a NSQIP database study. Laryngoscope Investig Otolaryngol. 2025;10:e70257. 10.1002/lio2.70257 PMC12442250 40969243

[15] Ottesen TD Malpani R Galivanche AR Zogg CK Varthi AG Grauer JN. Underweight patients are at just as much risk as super morbidly obese patients when undergoing anterior cervical spine surgery. Spine J. 2020;20:1085–95. 32194246 10.1016/j.spinee.2020.03.007 PMC7380546

[16] Zarrin H Vargas-Torres C Janevic T Stern T Lin MP. Patient sociodemographics and comorbidities and birth hospital characteristics associated with postpartum emergency department care. JAMA Netw Open. 2023;6:e233927. 36943266 10.1001/jamanetworkopen.2023.3927 PMC10031389

[17] Vester S Muhr A Meier J Süß C Kummer P Künzel J. Prehabilitation of dysphagia in the therapy of head and neck cancer- a systematic review of the literature and evidence evaluation. Front Oncol. 2023;13:1273430. 38188284 10.3389/fonc.2023.1273430 PMC10766849

[18] Gomes MCMF Ferreira PMV Almeida ACSM. Dysphagia, nutritional status, and quality of life in patients with head and neck cancer undergoing radiotherapy alone or combined with chemotherapy: an observational study. BMC Cancer. 2025;25:416. 40055649 10.1186/s12885-025-13695-y PMC11887319

[19] Parsons A Dewan K. Dysphagia and dysphonia after head and neck cancer. Oral Dis. 2025;31:2753–60. 39370743 10.1111/odi.15152

[20] Nosrat C Sibih Y Vallejo A Swarup I. Perioperative nutritional optimization in complex pediatric hip and spine surgery: a systematic review. J Pediatr Orthop. 2026;46:e181–6. 41104946 10.1097/BPO.0000000000003135

